# P-1469. Clinical Impact of Adherence to an Institutional Guideline for Multi-Drug Resistant Gram-Negative Infections

**DOI:** 10.1093/ofid/ofae631.1639

**Published:** 2025-01-29

**Authors:** Alyssa Cox, Sarah Minor, Amy L Carr

**Affiliations:** AdventHealth Orlando, Orlando, Florida; AdventHealth Orlando, Orlando, Florida; AdventHealth Orlando, Orlando, Florida

## Abstract

**Background:**

After publication of the IDSA Guidance on the Treatment of Antimicrobial Resistance Gram-Negative Infections, AdventHealth Central Florida implemented institutional pathways for treating MDR Gram Negative Infections. This study aimed to assess patient outcomes based on adherence or nonadherence to institutional pathways for MDR gram-negative infections.

**Figure:**
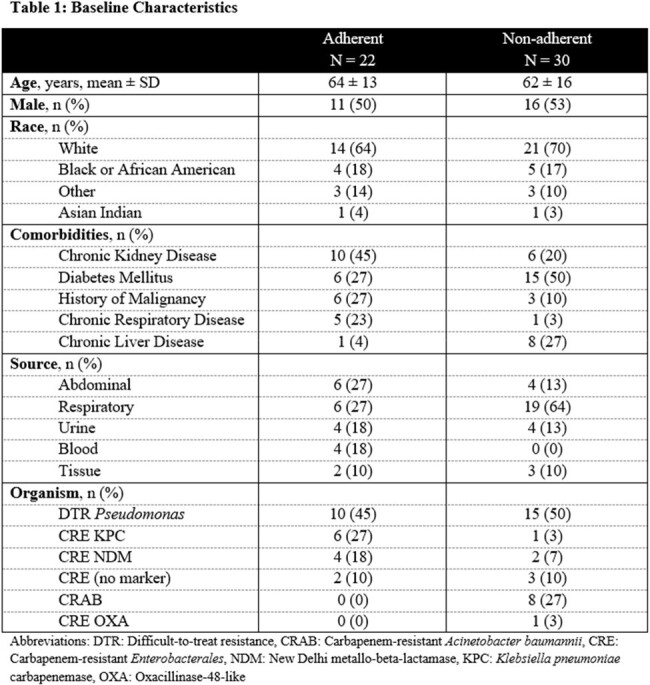

**Methods:**

This single center retrospective study reviewed hospitalized patients from August 2022 to April 2023. Patients were included if they were at least 18 years of age, hospitalized for at least 72 hours, and had an infection included in the institutional pathways for MDR Gram Negative Infections. Patients were excluded if they had multiple MDR gram-negative organisms or an extended-spectrum beta-lactamase-producing Enterobacterales. Data was collected by manual chart review. Cases which received antimicrobial therapy (drug and dose) aligned with the institutional pathway were classified as adherent. Statistical analysis were performed using StataSE.

**Figure:**
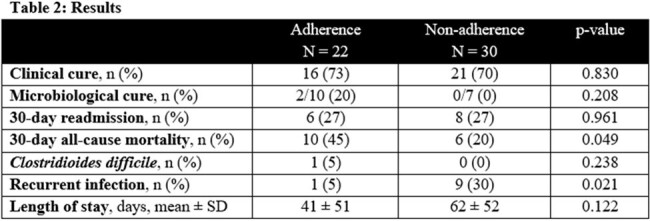

**Results:**

Of 1510 patients identified, 52 cases were eligible for inclusion (adherent: n = 22, non-adherent: n = 30). Patient characteristics were similar between groups (Table 1). The most common source of infection was abdominal or respiratory. Difficult-to-treat *Pseudomonas* was the most common pathogen. The primary outcome, clinical cure, was achieved in 22 (73%) patients in the adherent group and 30 (70%) patients in the non-adherent group (p = 0.830). Outcomes were similar for microbiologic cure, 30-day readmission, length of stay, and incidence of *C. difficile* infection (Table 2).

Mortality was higher in the adherent group (45% vs 20% p = 0.049) and there were fewer recurrent infections in the adherent group (5% vs 30% p = 0.021). These findings may be attributable to differences in infection source, source control, or severity of illness which were not evaluated in this study. Results support future investigation of pathway-guided care in larger scale study with a comprehensive review of factors influencing antimicrobial selection and clinical outcome.

**Conclusion:**

This review of hospitalized patients with MDR gram-negative infections supports the need for larger scale studies to determine if adherence to pathway-guided care can impact clinical outcomes.

**Disclosures:**

**Amy L. Carr, PharmD, BCIDP**, Entasis: Advisor/Consultant|Ferring: Advisor/Consultant|Gilead: Advisor/Consultant|InflaRx: Advisor/Consultant|LaJolla: Advisor/Consultant|Melinta: Advisor/Consultant|MicroGenDx: Advisor/Consultant|Shionogi: Grant/Research Support

